# High frequency of microdeletion in *TTY2* gene family in peripheral blood leukocytes of non-obstructive azoospermia patients

**DOI:** 10.3934/genet.2017.4.202

**Published:** 2017-12-15

**Authors:** Farideh Zonozi, Hossein Mozdarani, Mahdieh Salimi, Sohail Mozdarani, Parvin Fallahi, Sahar Mozdarani, Zahra Heidari

**Affiliations:** 1 Department of Genetics, Islamic Azad University, Damghan Branch, Damghan, Iran; 2 Department of Medical Genetics, Faculty of Medical Sciences, Tarbiat Modares University, Tehran, Iran; 3 Department of Medical Genetics, Medical Biotechnology Institute, National Institute of Genetic Engineering and Biotechnology, Tehran, Iran; 4 Cytogenome Medical Genetics Laboratory, Chamran Medical Building, Ale-Ahmad Highway, Tehran, Iran; 5 Infertility Center, Shariati Hospital, Tehran, Iran

**Keywords:** non-obstructive azoospermia, Y-chromosome microdeletion, *AZFc*, *TTY2* genes

## Abstract

About 10–15% of non-obstructive azoospermia (NOA) patients show *AZFc* microdeletion in their blood leukocytes. However, if *AZF* genes were involved in impaired spermatogenesis, a higher frequency of chromosomal microdeletions was expected. In this study the frequency of *AZFc* microdeletion was compared with *TTY2* gene family, i.e., *TTY2A2A* and *TTY2A12A* in blood leukocytes of NOA patients and normal fertile control. In the present study 30 normal fertile individuals with mean age of 35.0 ± 6.0 and 30 NOA patients with mean age of 34.0 ± 7.0 were screened for microdeletion of *TTY2L2A* and *TTY2L12A* at Yq11 and Yp11 respectively and sequence-tagged site (STS) markers for *AZFc* gene using multiplex PCR technique. At the first step karyotyping was done for all subjects using standard G-banding technique to identify patients with normal karyotype as well as non-affected normal controls for molecular analysis.

Results showed no *AZFc* microdeletion in normal and NAO patients whereas one *TTY2L2A* microdeletion in normal control (3.3%) and 4 in NOA (13.3%) was observed (*p* < 0.05). However our data indicated that 6 of 30 NOA patients (20%) showed *TTY2L12A* microdeletion whereas there was no observed microdeletion in normal control (*p* < 0.01).

Results indicate that the studied genes might be involved in impaired spermatogenesis more effective than the routinely screened *AZF* genes in infertile men. Therefore, screening these genes along with *AZF* genes might be valuable for infertile patients. The reason why these genes are deleted from Y chromosome is not known but might be associated with genomic instability induced by environmental physico-chemical genotoxic agents.

## 1. Introduction

Variety of factors are involved in male infertility; genetic abnormalities comprise about 30–40% of cases [1]–[3]. Genetic factors involved in male infertility may be consequent of Y or other chromosomes' microdeletions [4]–[8]. Higher range of chromosomal abnormality is estimated in infertile male (range from 2–28%) compared with general population (about 1%) [3],[9]–[11]. Klinefelter syndrome is one of the main causes of male infertility among all other structural and numerical chromosomal abnormalities [3],[11].

Y chromosome microdeletions are considered as the most frequent genetic causes of male infertility [6],[12]. It is estimated that over 2000 genes are involved in spermatogenesis cycle and occurrence of any mutational and chromosomal events in this process might lead to male infertility [7].

To date, deletions of AZoospermia Factors (*AZFs*) on the long arm of the Y chromosome (Yq) is considered as the second most frequent and widely studied genetic cause of male infertility after the Klinefelter's syndrome [13],[14]. *AZFs* genes located in Yq11 are classified as *AZFa*, *AZFb* and *AZFc* [15]. Although Y chromosome microdeletions are causally related to spermatogenesis defects, previous studies reported great variation, ranging from 1–55% in the percentage of infertile men carrying Y deletion. [16]–[18]. These significant variations in deletion frequency could be attributed to the location and extent of deletion. Moreover, different study designs, patients demography, quality of diagnostic settings, or inter-individual variations in the population might have been the cause of these variations [18],[19].

In azoospermic men, *AZFc* is the most commonly deleted interval (ranges from 10–15%), however, the frequency is lower for severe oligozoospermia men (ranges from 5–10%) [20],[21]. In Iranian population, the Y microdeletion frequency has been reported lower than worldwide reports and with a great variation [22],[23]. Because of the importance of *AZF* genes in male infertility, screening of the Y chromosome microdeletion of *AZF* genes is recommended in the diagnostic work-up of infertile men which is mainly done using polymerase chain reaction (PCR) on blood leukocytes [24].

Recently other microdeletions of Y chromosome, i.e., *TTY2* gene family with a different frequency of occurrence [25] are reported to be involved in male infertility [26],[27]. Pseudogene family *TTY2* is a Y-linked multicopy gene family with unknown function that includes *TTY2L12A* and *TTY2L2A* located at short arm (Yp11) and long arm (Yq11) of Y chromosome respectively [25]. It has also been shown that no similar sequences are on X or other autosome chromosomes [25]. Genes located on Y chromosome and expressed in testis are likely to be involved in spermatogenesis.

As known, sperm DNA damage is clearly associated with male infertility and abnormal spermatogenesis [28]–[30]. DNA damage is a prime cause of genome instability in cellular system. Genomic instability in azoospermia factor (*AZF*) region of Y chromosome especially in the *DAZ* genes was shown previously in lymphocytes of men exposed to low level ionizing radiation [31],[32] and in *in vitro* irradiated leukocytes [33]. It is possible that the microdeletions seen in genes involved in spermatogenesis are due to genome instability and of *de novo* origin after conception during embryogenesis. Therefore the aim of this study was to evaluate the frequency of microdeletion in *TTY2* genes versus *AZFc* in peripheral blood leukocytes of NOA patients.

## 2. Patients and methods

### 2.1. Study subjects

This experimental study was performed on whole blood samples obtained from 30 non-obstructive azoospermia patients and 30 normal men candidate for Assisted Reproductive Technology (ART) referred to Fertility and Infertility Center of Shariati Hospital (Tehran, Iran). Normal and patients' demographic data is presented in Table 1. In all cases, after three days of sexual abstinence, semen samples were collected by masturbation into sterile containers and were delivered to the laboratory immediately after ejaculation. A semen profiles were then performed and samples were classified according to World Health Organization criteria [34] into two groups (normal, azoospermia). Those samples considered as normal were obtained from men referred to IVF clinic because of their spouse infertility (female infertility). Whole blood was obtained by venipuncture in heparin and anti-coagulant ethylene diamin-tetraacetic acid (EDTA) containing tubes. The study was approved by the Ethical Committee of the Faculty of Medical Sciences of the Tarbiat Modares University (Tehran, Iran). All donors completed a written questionnaire to obtain information related to their life style, medical history and exposure to ionizing radiation, antibiotic consumption and gave their informed written consent for blood donation. Therefore, all samples had been screened to exclude individuals with smoking habits, varicocele, infections, hepatitis, HIV antibodies and radiation exposure.

**Table 1.** Demographic characteristics of subjects screened for AZFc, *TTY2L2A* and *TTY2L12A* microdeletions in studied groups.

Study groups	Normal individuals	Azoospermia patients
Number of subjects	30	30
Mean age ± SD (Years)	35.0 ± 6.0	34 ± 7.0
Mean BMI ± SD	27.3 ± 4.5	26.3 ± 4.8
Mean period of Infertility ± SD (Years)	1.4 ± 3.0	7.2 ± 5.3
Mean sperm count ± SD (×10^6^/mL)	76.3 ± 22.3	<0.1 ± 0.1
Total AZFc microdeletion (Mean ± SD)	0 (0.0 ± 0.0)	0 (0.0 ± 0.0)
Total *TTY2L2A* microdeletion (Mean ± SD)	1 (0.03 ± 0.18)	4 (0.13 ± 0.30)*
Total *TTY2L12A* microdeletion (Mean ± SD)	0 (0.0 ± 0.0)	6 (0.20 ± 0.40)**
Total *TTY2* microdeletion)	1 (0.03 ± 0.18)	10 (0.33 ± 0.47)**

* Significantly different with normal *p* < 0.05

** Significantly different with normal *p* < 0.01

### 2.2. Karyotype analysis

Karyotype analysis was performed to exclude those individuals with chromosomal abnormalities especially those involved in male infertility such as Klinefelter's syndrome from molecular analysis. Whole blood obtained from normal individuals and non-obstructive azoospermia patients in heparinized tubes was cultured in RPMI-1640 supplemented with 10% fetal bovine serum (FBS), antibiotics (penicillin (100 U/mL)/streptomycin (100 µg/mL), and 0.1 mL phytoheamaglutinin (all materials from Gibco BRL). Cells were exposed to thymidine 17 hours prior to colcemid treatment at final concentration of 0.4 µg/mL one hour before harvesting. Cells were harvested at 72 h after culture initiation. Harvesting, slide preparation and G-banding were performed according to routine standard procedure. At least 20 metaphases were analyzed for each subject. The resulting karyotypes were described according to the norms established by the International system for Human Cytogenetic Nomenclature [35]. Those patients with normal karyotype were enrolled for molecular analysis.

### 2.3. Screening for microdeletions in blood samples

**Figure 1.**
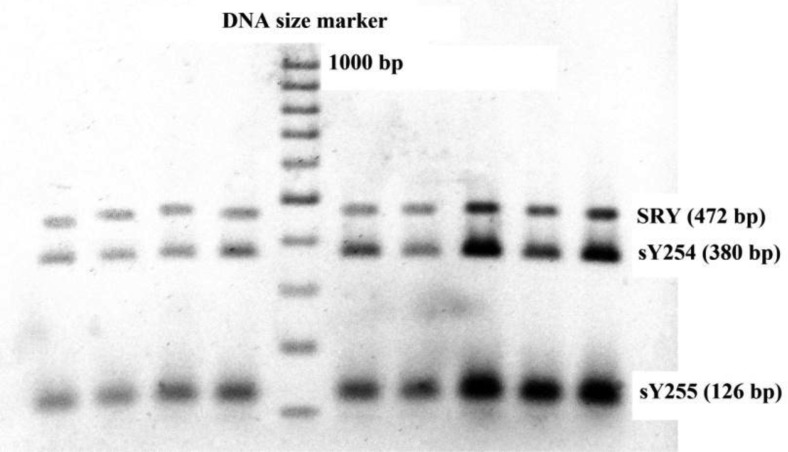
Sample gel run after multiplex PCR for *AZFc* microdeletion detection.

**Figure 2.**
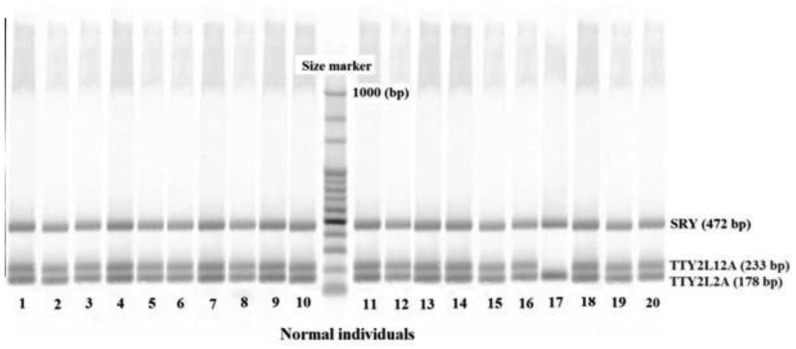
Mutiplex PCR showing the situation of *SRY*, *TTY2L12A* and *TTY2L2A* in leukocytes of 20 normal individuals.

**Figure 3.**
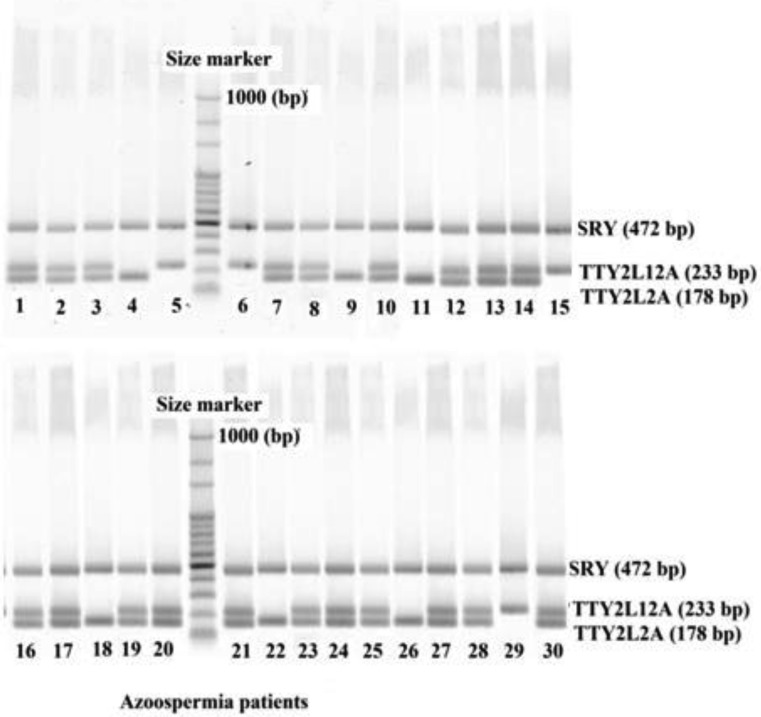
Mutiplex PCR showing the situation of *SRY*, *TTY2L12A* and *TTY2L2A* in leukocytes of azoospermia patients.

The patients and normal individuals with 46,XY, normal karyotypes were selected for *AZFc* and *TTTY2* microdeletion analysis. Peripheral blood samples were collected in tubes containing EDTA from cytogenetically normal infertile patients. All tests were performed on genomic DNA that was extracted from peripheral blood leukocytes using a commercially available DNA-isolation kit for mammalian blood (Cinna Puregene, Iran). For each participant, *AZFc*-specific STSs that spanned the sub regions (sY254 and sY255) as well as STS for *SRY* (Metabion, Germany) as internal controls were used to amplify specific regions of the Y chromosome using multiplex PCR [36]. Similarly specific STSs were used for *TTY2L12A* and *TTY2L2A* regions with *SRY* (Metabion, Germany) as internal control. The primers used in this study have been previously used by Yapijakis et al. [26]. Moreover, a blast search of the primer sequences showed that they were specific for the intended targets. Primer sequences used in this study are shown in Table 2. The PCR amplification comprised a total volume of 25 µL, containing 200 ng of genomic DNA; and standard PCR reaction mixture (All materials from Fermentas). Thermocycling program is modified for the multiplex PCR performed in this study. The PCR reaction products were separated on 2% to 3% agarose gels, and visualized by staining the gel with Syber safe DNA Gel stain (Invitrogen). Negative and positive samples were judged based on a negative and positive control. Figure 1 shows *AZFc* and Figures 2 and 3 *TTY2L2A* and *TTY2L12A* gel electrophoresis images in normal and azoospermia patients respectively.

### 2.4. Statistical analysis

Statistical analysis was done using SPSS software (version 19.0, Chicago, IL. USA). The Mann-Whitney *U*-test as well as one way analysis of variance (ANOVA) was used to compare differences between the study groups. Sigma Plot (2004) for Windows was used to draw figures, *p* < 0.05 was considered as statistically significant.

**Table 2.** Genes studied with their primer sequences and product size.

Gene	Sequence	Optimum Annealing Temperature (°C)	Product size (bp)
*SRY*	Forward: 5′-GAATATTCCCGCTCTCCGGA-3′	58	472
	Reverse: 5′-GCTGGTGCTCCATTCTTGAG-3′		
*TTY2L2A*	Forward: 5-CCTATCTGAGCAGGTACTTTAC-3′	58	178
	Reverse: 5-GTGTCATCTGTCTTTCTCAGTG-3′		
*TTY2L12A*	Forward: 5′-CAGACTGTGAGTTGGTTCTG-3′	58	233
	Reverse: 5′-TATGTGAGAGAGACCCTGTG-3′		
sY254	Forward: 5′-GGGGTTACCAGAAGGCAAA-3′	58	380
	Reverse: 5′-GAACCGTATCTACCAAAGCAGC-3′		
sY255	Forward: 5′-GTTACAGGATTCGGCGTGAT-3′	58	126
	Reverse: 5′-CTCGTCATGTGCAGCCAC-3′		

## 3. Results

**Figure 4.**
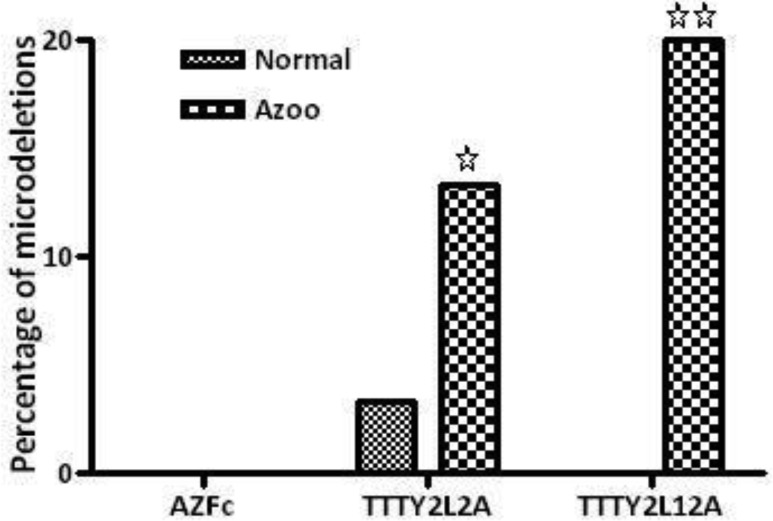
Percentage of microdeletions in genes studied in genomic DNA of normal and azoospermia patients, where no bar is shown the value is zero. (^☆^ Significantly different with normal *p* < 0.05. ^☆☆^ Significantly different with normal *p* < 0.01.)

Results are summarized in Table 1 and shown in Figure 4. As shown in Table 1, there was no significant difference between mean age and BMI of normal and patients studied in this project. However, significant difference shown for mean sperm count and mean period of infertility (*p* < 0.001) clearly indicates the status of fertility in both groups. Both normal individuals and non-obstructive azoospermia patients did not show any microdeletions in their *AZFc* reigon (Figures 1 and 4). No deletions in *TTY2L12A* gene were detected in fertile controls group. However, one person from control group had deletion in *TTY2L2A* (3.3%). In non-obstructive azoospermia group six patients showed deletion in *TTY2L12A* (20%), whereas four patients had deletions in *TTY2L2A* (13.3%). In addition, none of the patients showed deletions in both studied *TTY2* genes simultaneously. Results obtained for *TTYA2L2A* and *TTY2L12A* microdeletion were significantly different compared to control fertile group (*p* < 0.05 and *p* < 0.01 respectively). There was no correlation between microdeletions and age of the patients.

## 4. Conclusions

In the diagnostic work-up of infertile men, screening of Y chromosome microdeletion usually in *AZF* region is mainly done with the use of PCR on blood leukocytes [24]. It is believed that deletion of these loci may lead to spermatogenic failure and therefore is associated with azoospermia and oligozoospermia [37],[38].

Among *AZF* genes, *AZFc* is the most commonly deleted interval in men with azoospermia or severe oligozoospermia [20],[39]. However in the outmost side, the blood leukocytes *AZFc* deletion frequency in azoospermia patients is reported to be 10–15%. Although, recent report indicates that a high frequency of microdeletions in *AZFc* region especially *DAZ* gene might occur *de novo* during spermatogenesis when sperm nuclei are studied [8],[40]. Genome instability in azoospermia factor (*AZF*) region of Y chromosome especially in the *DAZ* genes was shown previously to occur in lymphocytes of men exposed to low dose and background level of ionizing radiation [31],[32] and in irradiated leukocytes *in vitro* [33]. As known, sperm DNA damage is clearly associated with male infertility and abnormal spermatogenesis [28]–[30]. Therefore, DNA damage in cells involved in spermatogenic cycle might contribute to mutational and chromosomal events leading to microdeletions in different genes involved in spermatogenesis.

As shown in Figure 4, control fertile individuals did not show microdeletions for *TTY2L2A* and *TTY2L12A* genes, expect one individual who exhibited microdeletion for *TTY2L2A*. The reason for this observation is not understood, but might be a *de novo* chromosomal alteration due to environmental exposure. Similar observation was previously reported for *DAZ* deletion in normal population exposed to high natural background radiation [31],[32]. The overall high percentage of *TTY2*-associated deletions observed in leukocytes of infertile azoospermia patients might suggests that *TTY2*-like transcripts may play a significant role in the complex process of spermatogenesis. As shown in Figure 4 azoospermia patients showed about 13.3 and 20 percent microdeletion for *TTY2L2A* and *TTY2L12A* respectively which is very high frequency compared to the overall frequency reported for *AZFc* microdeletion and more importantly in those patients with no deleted *AZFc*. These results might clearly indicate the involvement of the studied genes in spermatogenesis failure and male infertility. Perhaps their implication in male infertility might be more important than *AZF* genes which are routinely screened. A similar frequency of microdeletions of *TTY2L2A* and *TTY2L12A* genes is reported in oligoazoospermia and azoospermia patients form Greek patients [26]. The authors of this paper also concluded the involvement of these genes in spermatogenesis. With a great surprise a more recent publication reported a very low frequency (about 2.2%) microdeletion for both *TTY2L2A* and *TTY2L12A* genes in azoospermia patients with no *AZFc* microdeletion and higher frequency of microdeletions in azoospermia patients with unkown *AZF* situation [27]. The frequency of reported microdeletions is much lower and in contradiction with our results (Table 1 and Figure 4) and previous report by Yapijakis et al. [26]. The authors also attributed these deletions to male infertility. The rational for patient selection is not clear. If the authors claim that the presence of microdeletions in *TTY2L2A* and *TTY2L12A* genes are related to *AZF* deletion, then only in a lower percentage of patients' deletion in *TTY2* related genes could be detected. Moreover the deletion in *TTY2L12A* gene has nothing to do with *AZF* genes because it is located on the short arm of Y chromosome. Another possibility of lower frequency reported by Shaveisi-Zadeh et al. [27] might be related to the studied population with a different ethnicity. However, study of *TTY2* gene family is in its early stage. There are only limited reports associating these genes with male infertility. Although the possible causes of different frequency of microdeletions reported for these genes has already been discussed but for implementing testing these genes in paraclinic along with other Y microdeletions, there is a need for more researches and more data from different populations with different ethnicity and geographic distributions. So that we could be able to make a meta-analysis from different reports to provide a frequency range of microdeletions for *TTY2* gene family for clinical use. As known for routinely screened Y microdeletions among infertile or subfertile men, there is a very big variation in the frequency of occurrence from 1–55% [16],[18]. In Iranian population, the Y microdeletion frequency, according to the papers published by different investigators, has shown variable ranges from 5% to 52% which is within the range that has been reported worldwide [22],[23],[41]. Therefore, similar situation might be possible for *TTY2* gene family leading us to observe these microdeletions with different frequencies.

Our observations regarding to *TTY2* genes is in line with our previous observation for *DAZ* microdeletion and indicate that there are hot spots in these genes that make them vulnerable to damage and eventually deletion from entire DNA in Y chromosome [33]. Therefore exposure of men to physicochemical agents capable of inducing DNA damage might lead to genome instability that may cause microdeletion of these genes and eventually male infertility [42].

In conclusion a high frequency of microdeletions observed in our study on non-obstructive azoospermia patients without *AZFc* microdeletion might clearly indicate involvement of *TTY2* genes in spermatogenesis failure and male infertility.
